# Associations of maternal and infant metabolomes with immune maturation and allergy development at 12 months in the Swedish NICE-cohort

**DOI:** 10.1038/s41598-021-92239-3

**Published:** 2021-06-16

**Authors:** Olle Hartvigsson, Malin Barman, Hardis Rabe, Anna Sandin, Agnes E. Wold, Carl Brunius, Ann-Sofie Sandberg

**Affiliations:** 1 Food and Nutrition Science, Department of Biology and Biological Engineering, Chalmers University of Technology, Göteborg, Sweden; 2 Institute of Environmental Medicine, Karolinska Institutet, Stockholm, Sweden; 3 Institute of Biomedicine, Department of Infectious Diseases, University of Gothenburg, Gothenburg, Sweden; 4 Department of Clinical Sciences, Unit of Pediatrics, Umeå University, Umeå, Sweden

**Keywords:** Metabolomics, Immunology, Biomarkers, Immunological disorders, Paediatric research

## Abstract

Allergic diseases are the most common chronic diseases in childrenin the Western world, but little is know about what factors influence immune maturation and allergy development. We therefore aimed to associate infant and maternal metabolomes to T- and B-cell subpopulations and allergy diagnosis. We performed liquid chromatography-mass spectrometry based untargeted metabolomics on blood plasma from mothers (third trimester, n = 605; delivery, n = 558) and from the umbilical cord (n = 366). The measured metabolomes were associated to T- and B-cell subpopulations up to 4 months after delivery and to doctor´s diagnosed eczema, food allergy and asthma at one year of age using random forest analysis. Maternal and cord plasma at delivery could predict the number of CD24^+^CD38^low^ memory B-cells (p = 0.033, n = 26 and p = 0.009, n = 22), but future allergy status could not be distinguished from any of the three measured metabolomes. Replication of previous literature findings showed hypoxanthine to be upregulated in the umbilical cord of children with subsequent asthma. This exploratory study suggests foetal immune programming occuring during pregnancy as the metabolomic profiles of mothers and infants at delivery related to infants’ B-cell maturation.

## Introduction

Allergic diseases such as atopic eczema, allergic rhinitis, asthma and food allergies are the most common chronic diseases among children in the Western world. Allergic diseases are caused by a dysregulated immune system and the development of allergies depends on a complex interaction between genetic and environmental factors^1^. IgE-mediated allergy involves a faulty activation of the adaptive immune system, resulting in allergen-specific helper T- and B-cells, production of IgE antibodies and generation of symptoms upon renewed encounter with the allergen^2^. The immune system starts to develop in utero and rapid changes can be observed during this period and during the first months of life^3^*.* Functional T-cells^4^ are produced in the thymus as early as ten weeks gestation^5^ and start to circulate in the fetus late in the first trimester^4,6^. The thymus is maximally active during infancy and early childhood^7^. Little antigen stimulation occurs in utero*,* and a majority of the circulating T-cell at birth will be of a naïve CD45RA^+^ phenotype, but this fraction declines with age^8^ as they encounter environmental antigens and naïve T-cells are activated into effector cells, and later into memory T-cells. B-cell precursors are produced in the fetal liver^9^ where they are found as early as seven weeks gestation^10^. During the second and third trimesters of pregnancy, foetal B-cell production settles in the bone marrow^9^ and then continues throughout life to release transitional immature B-cells into the circulation^11,12^. These immature B-cells mature into naïve B-cells in the spleen^11^. Encounters with specific antigens and activation of naïve B-cells lead to the differentiation into either plasma cells that produce antibodies or memory B-cells that express the surface markers CD27 and CD24, but low or no CD38^13,14^. At birth, most B-cells in the circulation are naïve or immature CD5^+^ B-cells, while approximately 1% are of a CD27^+^ memory B-cell phenotype^15^. Higher proportions of immature CD5^+^ B-cells at birth have been associated with an increased risk of food allergy, atopic dermatitis and asthma^16,17^. However, what factors influence the maturation of immature B-cells and the activation of naïve B- or T-cells in utero are still uncertain.

Heredity is an important risk factor for the development of allergy^18,19^, while environmental factors such as older siblings^20,21^, growing up in a farming environment^22,23^ and pet ownership^19,24^ have been shown to be protective. According to the hygiene hypothesis^21^, exposure to microorganisms in early life stimulates the immune system in a manner that facilitates the development of tolerance to harmless foreign proteins^21^. Dietary factors also influence the risk of allergy development, consumption of fish^25^ and milk products^26^ by the pregnant mother being protective, and margarine appearing to increase the risk of allergy in the offspring^23,27^. Many diet-associated compounds rapidly appear in circulation after absorption in the intestine^28,29^. Epidemiological studies suggest maternal asthma to have a stronger influence on childhood asthma than paternal^19,30,31^, indicating that not only genetics are involved, but also other interactions in the mother–child interface, possibly including imprinting of the fetal immune system by metabolites in the maternal circulation. As several factors seem to relate to a maternal influence on the childs’ future allergies, we hypothesize that some of the risk of developing future allergic diseases could be associated with metabolites transferred from the mother to the child.

Metabolomics aims to provide comprehensive coverage of the metabolome (metabolic profile), i.e., the small-molecule metabolites (< 2000 Da) in a biological specimen (e.g., plasma or urine). Metabolomics lies furthest down in the ‘omics’-chain following genomics, transcriptomics, and proteomics. It gives a snapshot pictures of what reactions have occurred in the body and what compounds have been absorbed into the circulation. Metabolomics thus represents the omics discipline closest to the phenotype level^32^. Liquid chromatography-Mass spectrometry (LC–MS) provides the most comprehensive coverage of the metabolome and can reflect changes in several different metabolic pathways and can also be used to assess different lifestyle factors (e.g., diet, exercise or pollutants)^33^.

Previous studies have highlighted the potential to associate the metabolome with the occurrence of allergic symptoms and their severity in individuals with manifest allergies, such as asthma, atopic dermatitis and food allergy^34–44^. While such studies can be useful for diagnosis and selection of treatment for the alleviation of symptoms, they can neither predict allergic diseases, nor give any indications regarding their etiology. Focusing on predictive biomarkers could help to identify individuals at risk before the onset of symptoms as well as to help identify targets for preventive strategies. In a previous pilot study in a smaller cohort, we have prospectively predicted allergy development from the umbilical cord plasma GC–MS/MS metabolome^45^. To extend this, we here investigate the cord blood metabolome as well as the maternal metabolome in a larger cohort. To the best of our knowledge, no other previous study has attempted to predict allergy development from the mothers' plasma metabolome at or before delivery.

The aim of this study was to determine if there are relationships of the plasma metabolomes from mothers taken in the early third trimester and at delivery, and from infant umbilical cords at delivery with subsets of infant immune cells up to 4 months after delivery. Furthermore, the study also aimed to investigate the capacity of the metabolomes to predict allergic outcomes (atopic eczema, food allergy and asthma) in the infant, and examine if biomarkers found in our pilot study^45^ could be replicated. In addition, the study investigated whether previously reported biomarker candidates of manifest disease could be observed in the metabolic profiles of the present study and prospectively associated with allergy.

## Results

### Characteristics of the study population

Background characteristics for the study participants are shown in Table 1. There were no differences between allergic and non-allergic infants regarding maternal age, maternal Body Mass Index (BMI), birth weight, gestational length, parity, caesarean section or paternal heredity. Children with allergic mothers were overrepresented in the allergic group (p = 0.019).

**Table 1:** Background characteristics for the study participants divided according to allergic diagnosis (healthy or allergic) at 12-month follow-up.

	All participants n = 507	Allergic cases n = 82	Non-allergic controls n = 425	p-value
Birth weight (g)	3570 (3260–3970)	3565 (3275–3995)	3575 (3260–3955)	0.808
Gestational length (days)	281 (275–288)	281 (275–288)	282 (275–288)	0.710
Parity (> 0)^b^	266 (53%)	46 (57%)	220 (52%)	0.504
Caesarean section	68 (13%)	13 (16%)	55 (13%)	0.595
Maternal age (years)^a^	30 (27–34)	31 (27–34)	30 (27–34)	0.865
Maternal BMI (kg/m^2^)^a^	24 (22–28)	24 (22–29)	24 (22–28)	0.640
**Allergy within the family**				
Mother	198 (39%)	42 (51%)	156 (37%)	0.019
Father	226 (45%)	35 (43%)	191 (45%)	0.798
Siblings	97 (33%)	19 (37%)	78 (31%)	0.4972

^a^Maternal age and BMI were assessed at admission to maternity clinics in the first trimester.

^b^Defined as either nulliparous or parous.

Results presented as median (25th–75th percentile) or n (%). P values were obtained with Mann–Whitney tests for continuous data and with Pearson's chi-squared test for categorical data.

### Maternal and infant metabolic profiles in relation to subsets of T- and B-cells in the infants during the first year of life

Subsets of T- and B-cells were measured by flow cytometry in fresh blood samples drawn from the infants at four time points from birth to four months of age, i.e., at birth, at 48 h, one month and four months of age. Associations for the 23 subsets of cells, measured at four time points, with the three metabolomes are summarised in Suppl. Table S5. Associations were observed between maternal and child metabolomes and B-cell subsets in the child, that matched our a priori criteria of Q2 > 0.2 (Table 2). In contrast, no associations were found between the metabolomes and T-cell subsets. Specifically, the memory B-cell counts at four months of age were associated with the maternal metabolome during pregnancy and delivery. Memory B-cells were identified in two ways, either by expressing the CD27 surface marker or by the CD24^+^CD38^low^ phenotype. Memory B-cells expressing the CD27 surface marker were associated with the maternal pregnancy metabolome. Memory cells of the CD24^+^CD38^low^ phenotype were associated both with the maternal metabolome at delivery and the infant cord blood metabolome (Table 2). CD5^+^ B-cells, which represent an immature form of B-cells that we have previously noted to be more numerous in infants who subsequently developed allergy^17^, were associated with the infant metabolome at delivery (Table 2).

**Table 2:** Associations of T- and B-cell phenotypes with the plasma metabolomes from the mothers (at third trimester and at delivery) and infants (umbilical cord).

Outcome	Number of samples	Q2	p-value^a^
**Cell population outcomes vs. maternal metabolome during pregnancy (week 28)**			
CD27^+^ memory B-cell count at 4 m	24	0.20	0.023
**Cell population outcomes vs. maternal metabolome at delivery**			
CD24^+^CD38^low^ memory B-cell count at 4 m	26	0.21	0.033
**Cell population outcomes vs. umbilical cord metabolome**			
CD24^+^CD38^low^ memory B-cell count at 4 m	22	0.24	0.009
CD5^+^ B-cell count at 4 m	22	0.23	0.037

^a^p-value from permutation test (n = 100).

Associations between B-cell populations and metabolome did not appear to be heavily influenced by single outliers (Suppl. Fig. S1).

### Metabolites associated with immune cell maturation

Metabolite features of interest selected by the MUVR algorithm relating to immune cell maturation (Table 2) are reported in Table 3, together with putative annotation and univariate correlation with its respective associated immune cell population. Correlations of features in each model available in Suppl. Fig. S2.

**Table 3:** Putative annotations of metabolites selected in relation to immune maturation parameters and their correlation with associated immune cell population.

Feature (mz@rt)	Annotation	Method	Pearson r	MSI level
**Maternal pregnancy plasma in relation to CD27**^**+**^ **memory B-cell counts at 4 months**				
‘RP377.74198@479.325	Unidentified Phosphatidylserine	HMDB (MS1)	-0.751***	3
RP589.49395@574.6313	Diglyceride (32:1)	HMDB + Sirius (MS2)	0.657***	3
RP212.171317@346.555	Unidentified carnitine	HMDB (MS1)	0.647***	3
RP730.11357@437.645	Unknown		-0.515*	4
**Maternal delivery plasma in relation to counts of CD24**^**+**^**CD38**^**low**^ **memory B-cells at 4 months**				
RP265.1185@178.6629	Unknown		-0.698***	4
RN263.10382@178.73063	Phenylacetylglutamine	HMDB + CSI:FingerID (MS2)	-0.635***	2
RP446.37427@441.2178	Triglyceride (45:7)	HMDB (MS1)	-0.617***	3
RN843.53007@506.17202	Unknown		-0.438*	4
**Umbilical cord plasma in relation to counts of CD24**^**+**^**CD38**^**low**^ **Memory B-cells at 4 months**				
RP312.161740@369.1786	Unknown		0.713***	4
RN286.14498@351.3176	Unknown		0.730***	4
RN263.10382@178.73063	Phenylacetylglutamine	HMDB + CSI:FingerID (MS2)	-0.579**	2
**Umbilical cord plasma in relation to counts of CD5**^**+**^ **B-cells at 4 months**				
RP1180.82198@513.2628	Unknown		0.771***	4
RN398.208237@370.4420	Unknown		-0.673***	4
RN397.205563@371.37	Unknown		-0.666***	4
RP445.125473@419.18997	Unknown		0.431*	4

*HMDB* Human Metabolome DataBase, *MSI* Metabolomics Standard Initiative, *mz* mass over charge ratio, *rt* retention time, *RP* Reverse phase Positive, *RN* Reverse phase Negative.

*p < 0.05, **p < 0.01, ***p < 0.001.

Only 6 of the 15 selected features of interest could be fully or partially identified: CD27^+^ memory B-cells correlated negatively with an unidentified phosphatidylserine and positively with an unidentified diglyceride (32 carbons, one unsaturation) in maternal pregnancy plasma. CD24^+^CD38^low^ memory B-cells correlated negatively with a triglyceride (45 carbon atoms, seven unsaturations) in maternal delivery plasma. Notably, CD24^+^CD38^low^ memory B-cell counts were correlated negatively with phenylacetylglutamine concentrations in both maternal and infant delivery plasma.

### Maternal and infant metabolic profiles in relation to allergy in the infants during first year of life

Allergic and non-allergic infants were matched in pairs. Classification rates (i.e., number of individuals classified in the correct group based only on the metabolic profiles) for the three metabolomes in relation to the allergy outcomes are shown in Table 4. The classification rates for infant allergies related to the maternal pregnancy metabolome ranged from 32% up to a modest 58%, i.e., at maximum 58% of the infants were classified correctly as cases or controls. The classification rate for models based on the maternal metabolome at delivery ranged between 33 and 61% and based on umbilical cord plasma metabolome from 32 to 42%. The best classification rate was achieved for any allergic diagnosis (i.e., eczema and/or food allergy) in relation to maternal metabolome, however still under our a priori limit of 66% for permutation testing (a classification rate of 50% corresponding to random guessing).

**Table 4:** Associations of allergic outcomes with the mothers' plasma metabolomes during the third trimester and delivery and from the umbilical cord at birth for matched case/control pairs.

	Maternal pregnancy metabolome		Maternal metabolome at delivery		Umbilical cord blood metabolome	
	n	Classification rate (%)	n	Classification rate (%)	n	Classification rate (%)
Asthma	34	32	30	33	38	42
Food allergy	33	58	31	56	48	40
Eczema	33	36	32	59	42	36
Any allergy	54	39	51	61	44	32

Results presented as the proportion of correctly classified samples as healthy or allergic using random forest multilevel modelling based on matched case–control pairs.

### Metabolites previously associated with manifest allergy

We selected metabolites for targeted analyses from 13 previously published studies ^34–37,39–43,45–48^ where associations between metabolites and allergic outcomes were found, 151 metabolites based on analysis of plasma, 20 on urine and 11 on exhaled breath. Of all the 182 metabolites that were associated with manifest allergy in these studies, we found five of them in cord plasma to be associated with future allergy in our study. We did not find any of these metabolites in the maternal plasma to be associated with allergy (Table 5). The only metabolite where an association was found to be in the same direction and for the same allergic disease as in the literature was hypoxanthine. The other four were found in relation to other allergic diseases or associated in the opposite direction to what was previously reported in the literature.

**Table 5:** Associations of prospective allergy diagnosis with metabolites previously related to manifest allergy.

Cord plasma metabolite^a^	Association in literature	Association in present study	Paired t-test		Generalized linear model	
			FC^b^	p-value	FC^b^	p-value
Histidine	Lower in asthma patients 18–63 years old^34^	Higher in subsequent food allergy	1.91	0.029	1.17	0.045
Hypoxanthine	Higher in asthma patients 9–19 years old^41^	Higher in subsequent asthma	5.13	0.053	1.54	0.030
Hydroxybutyric acid	Higher in atopic dermatitis patients 6–10 months old^39^	Lower in subsequent atopic dermatitis	0.93	0.092	0.77	0.025
Tyrosine	Higher in children with asthma 6–14 years old^48^	Lower in subsequent atopic dermatitis	0.94	0.090	0.80	0.005
Proline betaine	Correlated with asthma severity in children 6–14 years^43^	Higher in subsequent atopic dermatitis	31.78	0.017	1.64	0.083

^a^All metabolite associations reported for cord plasma. No associations were observed with the maternal plasma metabolites.

^b^Data presented as fold change (FC), with FC > 1 indicating a positive association with allergy.

## Discussion

The aim of the present study was to investigate whether T- and B-cell immune maturation during infancy and early allergy development were related to maternal and fetal metabolomes. Plasma was obtained from maternal blood obtained at week 28 of pregnancy and at delivery and from umbilical cord blood. We found associations between B-cell maturation and maternal as well as fetal metabolomes. The CD24^+^CD38^low^ B-cell count in four months old infants was moderately associated with maternal and infant metabolomes at delivery. Further, the maternal pregnancy metabolome was associated with the concentration of CD27^+^ B-cells in 4 months old infants. Both the CD24^+^CD38^low^and the CD27^+^ phenotypes are markers of memory B-cells.

The multivariate MUVR models selected 15 metabolites that were related to immune maturation in any of the three metabolomes. Only one of these could be completely identified; phenylacetylglutamine (PAG) whose levels in both maternal and infant plasma at delivery negatively correlated with circulating memory CD24^+^CD38^low^ B-cell counts in the infant at four months of age. PAG is a gut-microbial metabolite formed in the colon^49,50^ but can also be produced by mitochondria in the human liver and kidney cells^51–53^. It is formed by decarboxylation of phenylalanine into phenylacetic acid followed by conjugation with glutamine into PAG. In mice, PAG has shown anti-inflammatory properties^54,55^ reducing the production of the cytokines TNFα and interleukin-6 (IL-6), the latter being an important stimulant and maturation factor for B-cells. This might explain why a higher concentration of PAG in both maternal and umbilical cord blood associated with lower memory B-cell count in the infant. We could not identify any specific metabolite responsible for the association between pregnancy metabolome and CD27^+^ memory cells in the infant at four months of age.

We also identified an association between the infant cord blood metabolome and CD5^+^ B-cells at four months of age. These cells are primarily found among so called "translational" B-cells, which are immature B-cells recently produced in the bone marrow (or fetal liver). Higher levels of CD5^+^ B-cells have previously been shown to lead to higher allergy incidence^16,17^ and are a sign of an immature immune system. Unfortunately, none of the metabolites related to this association could be identified.

We further investigated whether allergic disease during the first year of life could be predicted by metabolomic profiles, measured in the mother during pregnancy and at delivery and in the infants' umbilical cord blood. We could not observe any prospective associations between any of the assessed metabolomic profiles with any of the allergic diseases diagnosed at 12 months of age; food allergy, eczema or asthma. Allergic diseases tend to appear in succession during childhood, with eczema and food allergy being early manifestions, followed by asthma that usually appears around school age and hay fever often presents in adolescence, a phenomenon termed the "atopic march"^56^. At 12 months, the full spectrum of allergic diseases have not manifested in many individuals and it might thus be too early for establishing a reliable diagnosis of other allergic manifestations than food allergy and eczema. Furthermore, some early atopic manifestations may be transient and disappear later during childhood. The infants examined here will be offered a second clinical examination at four and six years of age as well. Possibly, associations between cord and pregnancy metabolomics and allergy at an older age might be revealed, which will be the focus of future studies.

Our results could be influenced by maternal heredity being more frequent in the allergic group, potentially contributing to confounding. It was, however, not feasible to stratify analyses according to maternal allergy due to the low number of allergy cases. Moreover, we did not find any associations between allergy and metabolome. Consequently, the null findings are likely not affected by potential differences in recruitment between general and high-risk populations.

Several studies have identified associations between metabolome and manifest allergic diseases such as asthma and eczema. The metabolites identified in these studies might also have potential as early (prediagnostic) predictors of allergic disease. To complement the exploratory random forest-based approach, we thus performed a targeted analysis of 182 metabolites previously related to allergy development, asking whether some of them were also prospectively associated with disease. Among all investigated associations, only hypoxanthine was regulated in the same direction in our study as in the literature: We found that higher levels of hypoxanthine in cord blood plasma associated with asthma at 12 months of age. It was previously reported to be higher in 9–19 years old children/adolescents with asthma compared with non-allergic controls^41^. Four other metabolites associated with manifest allergic disease in previous studies, were either not associated with the same allergic disease in our study, or associated in the opposite direction. It should be noted that these associations represent exploratory analyses not adjusted for multiple comparisons and these results should be interpreted with caution.

Previous studies have found metabolic profiles to be associated with manifest allergic disease, including asthma^34,42^ and atopic dermatitis^35,39^ in adults and food allergy^40^, asthma ^37,38,40,41,46–48^ and atopic dermatitis^36^ in children. Still, to the best of our knowledge, our study is the first to prospectively analyse the metabolome before the onset of disease. The discrepancy between the results from our study and previous studies may be due to several reasons. Previous studies have been cross-sectional with metabolic profiles measured in samples collected after onset of the disease. Therefore it is uncertain whether potential metabolite biomarkers from those studies are related to the cause, mechanisms or symptoms of disease, or even from medication or reverse causation from lifestyle interventions. However, our study employed a prospective design, where symptoms or medication likely should not interfere to an appreciable extent. Further, previous studies have mainly been performed on adults and children older than eight years. In contrast, we have focused on immune maturation and allergy development before and during delivery and in early life. In addition, previous studies have investigated the metabolome predominantly in urine, both in relation to childhood asthma^47,48^ and atopic dermatitis in infancy^39^. Other studies have used less common biospecimen that reflects the phenotype of the specific allergic disease in question, such as exhaled breath condensate for asthma^37,38,57^ and non-lesioned skin for atopic dermatitis^58^. However, we have investigated the metabolomes in plasma as we expected this to best reflect what is being transferred to the fetus in utero as well as during birth. These differences in biospecimens may also contribute to the lack of replication for a majority of the reported metabolite markers from the literature.

A major strength of this study is that allergy diagnosis was performed through consistent, well-documented procedures by the same allergology specialist, thus improving accuracy compared to self-reported questionnaires. However, although the occurrence of allergic diseases during the first year of life is well documented in our study, the infants continue to develop allergies later in life and the control group may in fact contain pre-allergic infants. In combination with the low proportion of allergic cases, this imposes limitations for discerning prospective associations between immune maturation, allergy development and the maternal and cord plasma metabolomes. Another strength of this study is that we used robust statistical methods to reduce the likelihood of false-positive discoveries in our main, machine learning-based analyses.

An important limitation of the current study is the long sample storage at 4 °C until centrifugation for samples collected from deliveries occurring during weekends, which may induce undesired, variability in the measured metabolome. We addressed this aspect by performing sensitivity analyses, excluding samples stored for longer than 24 h. These analyses showed similar results, suggesting that pre-analytical management was not a main contributor to the null findings in our main analyses. In addition, associations may have been diluted from variability inherent to untargeted LC–MS metabolomics. However, data sanity checks (regression on BMI for the two maternal metabolomes and gestational length for the umbilical cord metabolome; Q2 > 0.2) indicated that the data was structurally sound.

Another limitation is the potential overfitting from the low number of samples for the cell count results as well as the multiple testing of outcome variables. However, the distribution in relation to the cell count variables indicate that overfitting was at least not due to a few outliers. As this is an explorative study, we only report nominal p values and results should be interpreted with appropriate caution.

Furthermore, the present study utilized reverse phase chromatography only, limiting the discovery of relevant metabolic features more easily observable from hydrophilic interaction liquid chromatography or lipidomic platforms. Still, the reverse phase has overlap with those complementary techniques, indicating that the null findings are likely robust for prediagnostic allergy development at one year of age.

It is important to note that the NICE birth cohort is not population-based. Due to extensive sampling of biological samples and questionnaire data, we were only able to include around 10% of the women that delivered at the Sunderby hospital during the inclusion period. This reduced both the power and the potential generalizability of results to the entire population.

## Conclusion

In this study, we found significant but modest associations between infant B-cell maturation and the plasma metabolic profiles from mothers during pregnancy and at delivery, as well as infants at delivery suggesting foetal immune programming in utero. However, since B-cell population measurements were only available in a smaller subset of the samples, our results should be interpreted with caution and need to be replicated in larger studies. We found no associations between the full metabolomes and allergy development up to 12 months of age; longer follow-up should provide further evidence regarding the potential to discover prospective biomarkers of allergy development.

## Materials and method

### Study population

The birth-cohort NICE (Nutritional impact on the Immunological maturation during Childhood in relation to the Environment) recruited pregnant women during 2015–2018 with planned birth at Sunderby Hospital, in northern Sweden. The study protocol has been described in detail before^59^. The study was approved by the Regional Ethical Review Board in Umeå (2013-18-31 M, 2016-232-32); written informed consent was obtained from the prospective parents. All methods were performed in accordance with the ethical approval and in accordance with the declaration of Helsinki.

According to predefined protocols, allergy was diagnosed by a pediatric allergologist (author AS) at 12 months of age: Food allergy was defined as an immediate or delayed reaction to intake of a specific food with improvement once the food was excluded from the diet. Except when the first reaction was acute and severe, the diagnosis was confirmed by a provocation causing similar symptoms. Sensitization or specific IgE antibodies against the particular food supported diagnosis but was not mandatory. Atopic dermatitis was diagnosed according to Williams' criteria^60–62^. Asthma was defined as any of the following: wheezing between infections, persistent wheeze for ≥ four weeks, wheezing during infection combined with concomitant allergic disease, or three episodes of wheezing during an infection, without concomitant allergic disease. In total, 539 children participated in the 12-month follow-up, 43 of whom were diagnosed with food allergy, 36 with atopic eczema and 35 with asthma^26^.

### Collection of blood samples

Blood was sampled in EDTA tubes (Becton Dickinson, New Jersey, USA) in gestation week 28, (participants were encouraged to fast for 8 h prior to this sampling), and from mothers and infants (cord blood) at delivery. In gestational week 28, samples were taken by midwives at maternity wards. The samples were left at room temperature for 30 min before centrifugation. Centrifuged tubes were stored at 4 °C until transportation to the research laboratory at the hospital the same day or for some samples the following workday. Upon arrival at the research laboratory, the plasma was aliquoted and frozen in − 80 °C. At delivery, venous blood was collected from the mothers. The umbilical cord was clamped and severed and blood was squeezed out into EDTA tubes. Samples were stored at 4 °C at the delivery ward, without centrifugation, until transportation to the research laboratory at the hospital. At weekends, transportation occurred the first following workday. At the research laboratory, the delivery samples were centrifuged and plasma was collected, aliquoted and stored at − 80 °C. The time elapsed from sampling until freezing for individual samples is shown in Suppl. Figs. S1–S3

**Figure 1:**
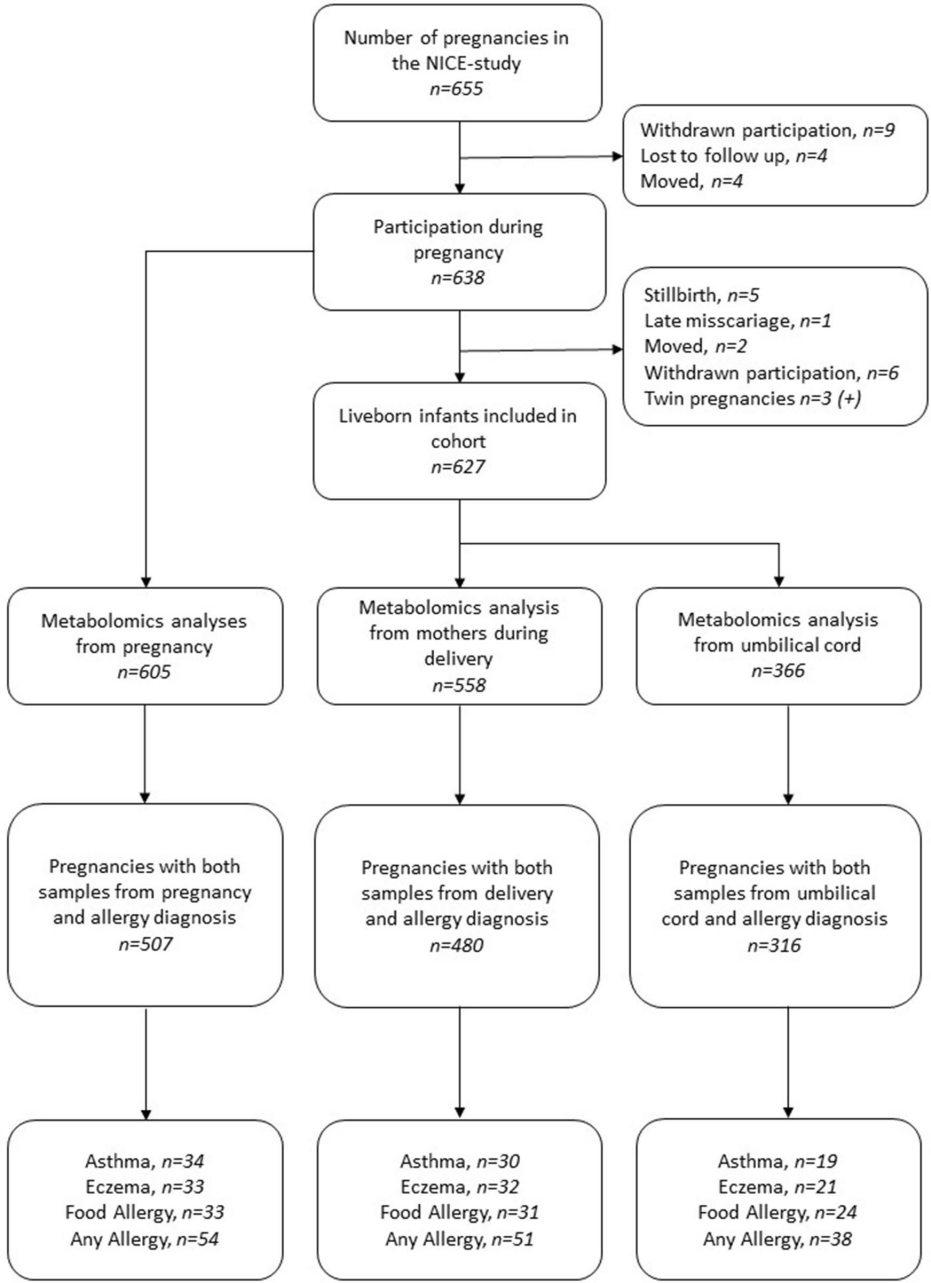
Overview of the number of cases and controls used in analyses. (+) indicating that three more children are added to the study due to twins being born.

In total, 605 samples from pregnancy, 558 maternal samples from delivery and 366 umbilical cord samples were available (Fig. 1).

### Metabolomics analysis

Prior to analyses, samples were randomly assigned into 20 batches. For quality control (QC) samples, 50 µl each of the 71 samples from the first batch were mixed and vortexed for 2 min. Aliquots (30 µl) were stored at – 80 °C.

The day before analysis, samples and QC were transferred to -20 °C. Samples were thawed at room temperature (RT) for 30 min, kept at 4 °C for 2 h and vortexed for 10 s. Aliquots (30 µl) were mixed with acetonitrile (200 µL, 4 °C) in 96-well plates that were sealed and put on an orbital shaker (1000 rpm, 3 min) and centrifuged (500×*g*, 4 °C, 20 min). After filtration (Captiva ND plates; Agilent P/N A5969045) using water vacuum for 5 min, 45 µl was retrieved and kept at 4 °C.

Untargeted LC–MS metabolomics was performed on an Agilent Infinity 1290 UHPLC with an Agilent 6520 QTOF mass spectrometer. Centroided MS data were acquired with Mass Hunter B.08.00. LC and MS settings are reported in Suppl. Table S1. Five QCs were injected before the first sample for each batch, and one QC after every 12 samples.

### Data preprocessing

Raw .d data files were converted to .mzML using Proteowizard (v 3.0.18345)^63^. All further computations were performed in R v 3.6.0^64^ at the Swedish National Infrastructure for Computing resource for sensitive data (SNIC-SENS). Data were processed using xcms v3.6.0^65^. Preprocessing parameters were obtained from manual and IPO-assisted optimization^66^ (Suppl. Table S2). Values missing after hard filling with fillChromPeaks (2.5% in positive and 13% in negative ionization) were imputed using an in-house PLS-based algorithm (mvImpWrap() function; https://gitlab.com/CarlBrunius/StatTools). Correction for intensity drift was performed using batchCorr v0.2.4^67^. Isotopes, adducts and fragments were aggregated using RAMClust v1.0.6^68^, resulting in 3296 features in positive and 789 features in negative mode (Suppl. Table S3) referred to by a unique combination of mass to charge ratio (mz) and retention time (rt). Scripts for parameter optimization and preprocessing are available from the authors upon request.

### Flow cytometry

Flow cytometry was performed on blood samples collected from infants at birth (cord blood) (n = 123), 48 h (n = 70), one (n = 93) and 4 months (n = 99) of age. Samples were stored dark in RT directly after collection and staining was performed within 48 h of sampling.

Whole blood (50 µl) was added to TruCount™ tubes (BD Bioscience, Erembodegem, Belgium), together with 20 µl antibody cocktail containing anti-CD4, anti-CD8, anti-CD20 and anti-CD45 (Suppl. Table S4) and incubated dark at RT for 15 min. BD Lysing Solution was added and allowed to act for 15 min. Samples were analyzed within 1 h of staining in an Accuri C6 (BD Bioscience). For each sample, 5000 beads were collected in the flow cytometer.

For B- and T-cell phenotype, 900 µl blood was lysed with RCB lysis buffer (eBioscience) (15 min, RT) and stopped with FACS buffer, before centrifugation (5 min, 300×*g*). The supernatant was discarded and the pellet resuspended with 1 ml FACS-buffer. Cell suspensions (50 µl) were stained with 30 µl antibody cocktail (Table S4) in 96 V-bottom plates for (20 min, 4 °C, dark), washed with 300 µl FACS-buffer and centrifuged (3 min, 300×*g*). The supernatant was discarded and the cells were resuspended in 300 µl buffer (Foxp3 Fixation/Perm. Kit, eBioscience) and incubated at RT for 15 min. Cells were then centrifuged (3 min, 500×*g*), washed with Foxp3 buffer, again centrifuged (3 min, 500×*g*), resuspended in FACS-buffer and stored in the dark (4 °C) until analysis in the Accuri C6 flow cytometer. Flow cytometry data were analysed using Flow Jo v10 (TreeStar, Ashland Oregon). Gating strategy for the FACS analyses are shown in Supplementary Fig. S4.

### Data collection

Maternal characteristics, such as age, educational level, parity and BMI, were collected from hospital records and data on heredity to allergic diseases, pet ownership and residence were collected by the study paediatrician at the 12-month follow-up.

### Data analysis

Multivariate Random Forest analysis (RF) with repeated double cross-validation and unbiased variable selection, MUVR package v0.0.971^69^, was used to associate metabolome data matrices with T- and B-cell subpopulation counts measured at birth, 48 h, one month and four months of age (further information on which subpopulations in Suppl. Table S5, and MUVR parameters in Suppl. Table S6). Significance was assessed using permutation tests (n = 100) on models with potentially relevant performance, defined a priori as classification rates > 66% or regressions with Q2 > 0.2, set to reflect meaningful predictions. As points of reference, Q2 = 0 and classification rate = 50% would represent null associations (random conditions). (Definition of Q2 and classification rates available in Supplementary Methods). Sensitivity analyses were performed by excluding samples stored for > 24 h (n = 130, 159 and 118 pregnancy, delivery and umbilical cord samples, respectively) before centrifugation.

The three metabolic matrices, i.e., maternal pregnancy and maternal and infant at delivery, were associated to asthma, atopic eczema and food allergy using RF classification. Allergic cases (Fig. 1) were matched to non-allergic and non-sensitized controls (1:1) based on gestational length (± 10 days), parity (previous children or not), mother's age (± 5 years), sex and maternal BMI (± 5). Matching for caesarean section could not be achieved. Matching criteria had to be relaxed for BMI (± 11, n = 4 cases) and gestational length (± 61 days, n = 7).

### Metabolite identification

Features of interest from the statistical modelling (still not annotated, but referred to by m/z and retention time) were selected for masspectrometric fragmentation (MSMS) analysis. Samples with high levels of these features were reanalysed using the same procedure as described above while specifically targeting these features for fragmentation. Annotation of features was performed using MSMS matching to the Human Metabolome DataBase (HMDB) and using CSI:FingerID^70^ and CANOPUS^71,72^. For features where MSMS data was unavailable, MS1 matching was attempted using HMDB.

### Metabolites previously associated with manifest allergy

W allergic disease could be e investigated whether previously reported possible metabolite biomarker candidates of manifest prospectively associated with allergy development in our study.

The biomarker candidates were identified first from a literature search, complemented by metabolomics studies in plasma and serum reported by Schjødt et al.^44^ (a complete list of investigated metabolites in Suppl. Table S7). We then matched all qualified features from our untargeted metabolomics experiment to plausible molecular masses obtained from the biomarker candidates (i.e., neutral monoisotopic masses, combined with the adducts [M + H] +, [M + Na] +, [M + K] +, [M + NH_4_] +, [M + CH_3_OH + H] +, [M + ACN + H] + and [M + 2H]2+ in positive mode and [M–H]–, [M–H_2_O–H]–, [M + Na–2H]–, [M + K–2H]–, [M + Cl]–, [M + FA–H]–, [M + HAc–H]– and [M–2H]2– in negative mode) within a mass tolerance of 10 ppm. Associations between allergic disease and metabolomic features matched to biomarker candidates were assessed using two approaches: first, paired t-tests were performed between matched case/control pairs. Second, since matching criteria might not have been sufficiently strict in order to fully match controls to cases, generalized linear models were performed with allergy as independent factor and metabolomic feature as dependent variable, further adjusted for gender, gestational length, cesarean section, age of mother, parity and BMI of the mother. Features, nominally significant in either of these two models, were further filtered out if not matching the suggested metabolite biomarker candidates, first by MS (if, e.g., matching to an isotope instead of the main fragment) and later by MSMS by comparing fragmentation patterns to the Human metabolome database hits of the suggested biomarker candidates.

**Figure 2:**
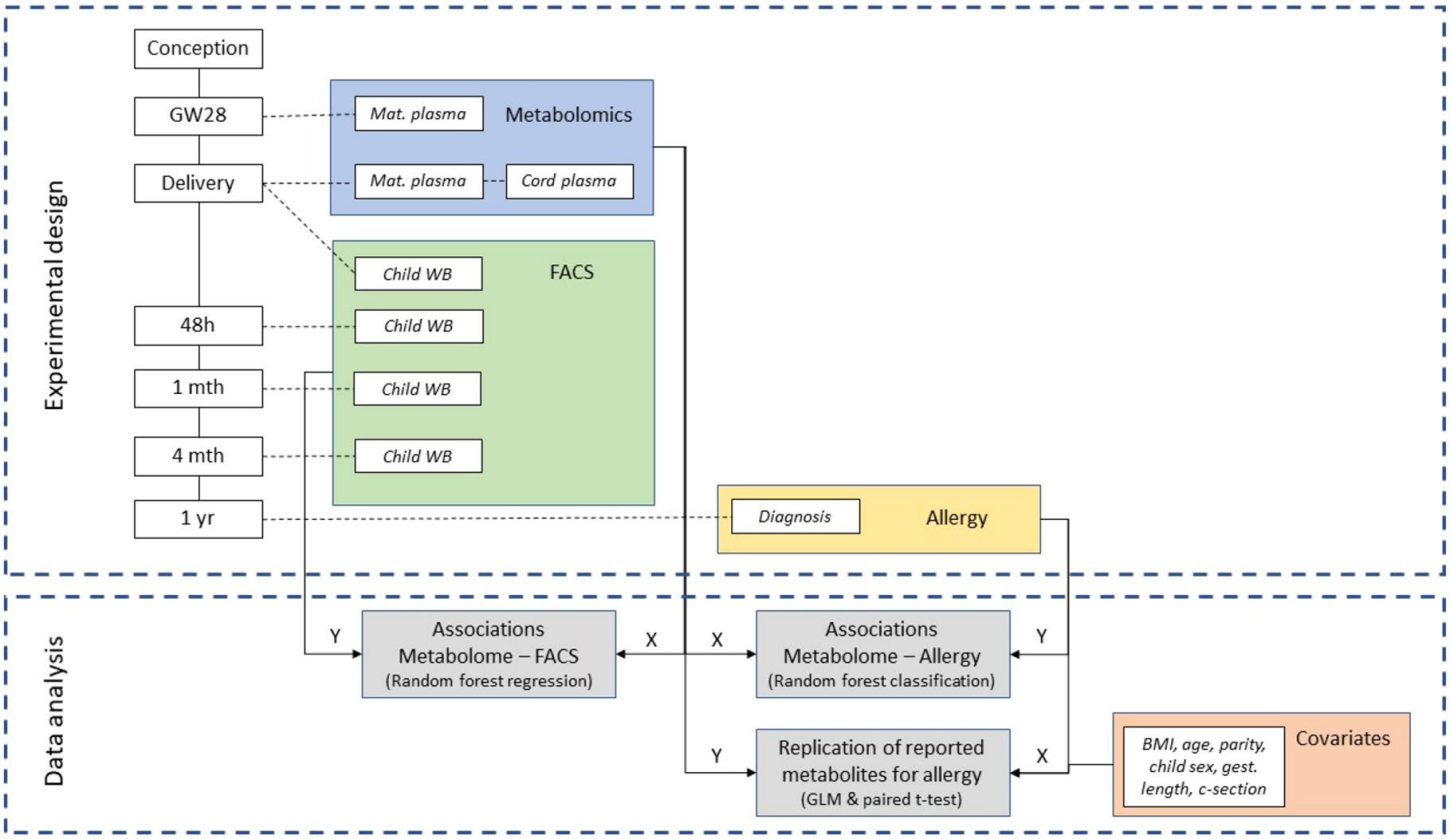
Timeline of sampling and analytical flow chart of data analysis.

A general overview of sampling and analytical work flow are shown in Fig. 2.

The datasets generated during and/or analysed during the current study are available from the corresponding author on reasonable request and fulfilment of ethical requirements.

## Supplementary Information

Supplementary Information.
