# Supplementary material for: Associations of maternal and infant metabolomes with immune maturation and allergy development at 12 months in the Swedish NICE-cohort
Source: Sci Rep. 2021 Jun 16;11:12706. doi: 10.1038/s41598-021-92239-3 (PMC8209090; doi:10.1038/s41598-021-92239-3)
Supplement: Supplementary file 1 — Supplementary Information. [file 41598_2021_92239_MOESM1_ESM.docx]

## Supplementary material


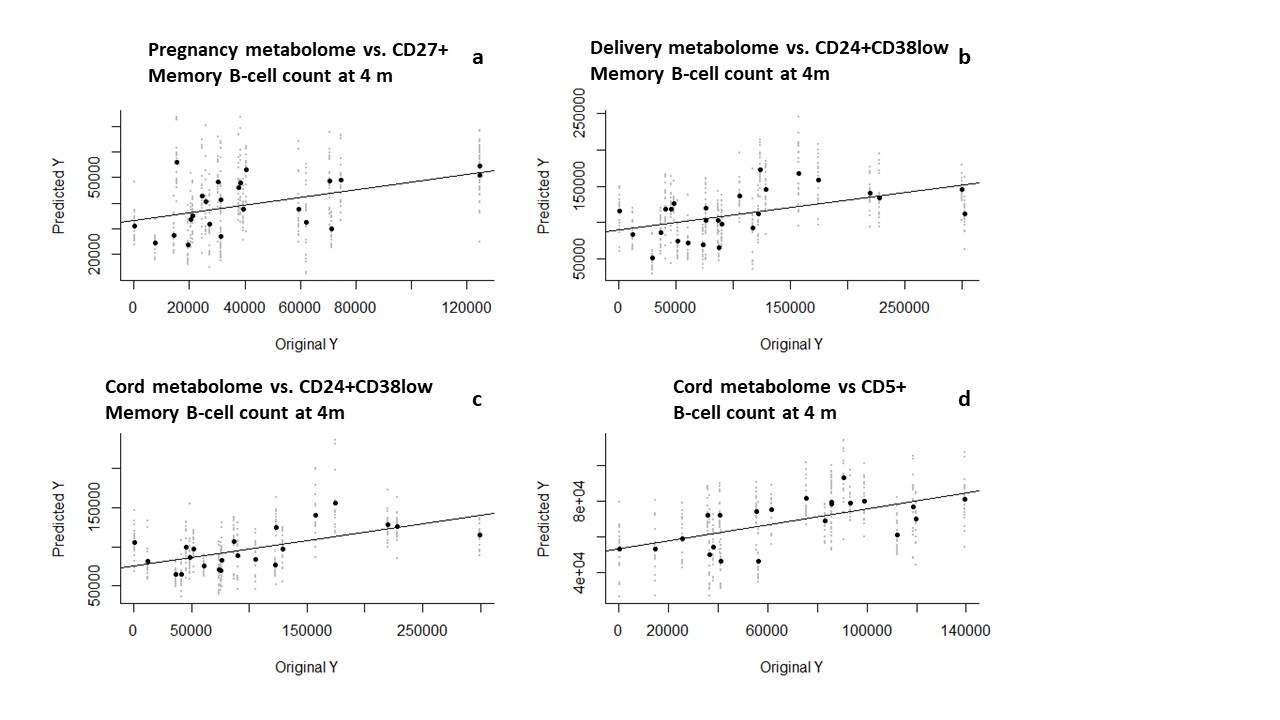


**Figure S1.** Predicted versus actual values for a) CD27^+^ Memory B-cell count at 4m vs pregnancy metabolome , b) CD24^+^CD38^low^ Memory B-cell count at 4m vs maternal delivery metabolome , c) CD24^+^CD38^low^ Memory B-cell count at 4m vs cord metabolome and d) CD5^+^ B-cell count at 4m vs cord metabolome obtained from random forest modeling. Small grey circles indicate predicted value from each iteration of the modelling and larger black circles indicate average predictions.

**
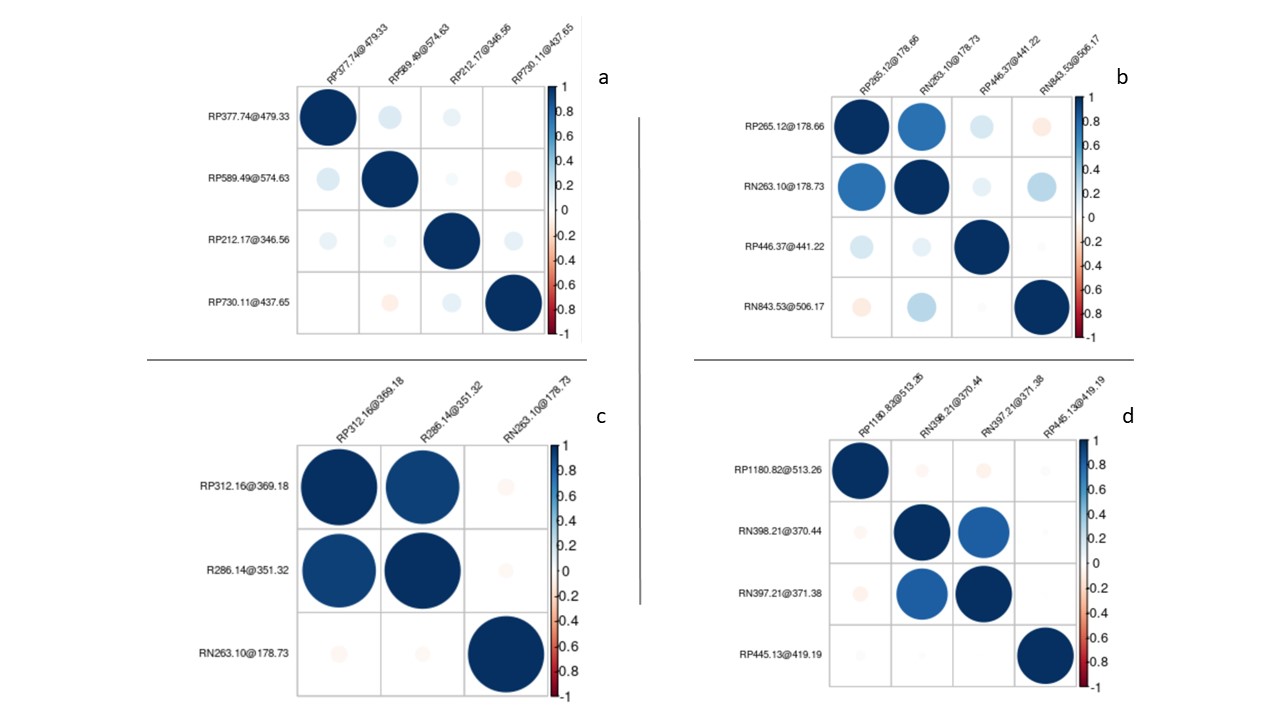
**

**Figure S2.** Correlations of all metabolites deemed to have a significant impact on modelling performance by the MUVR algorithm for a) CD27^+^ Memory B-cell count at 4m vs pregnancy metabolome , b) CD24^+^CD38^low^ Memory B-cell count at 4m vs maternal delivery metabolome , c) CD24^+^CD38^low^ Memory B-cell count at 4m vs cord metabolome and d) CD5^+^ B-cell count at 4m vs cord metabolome.


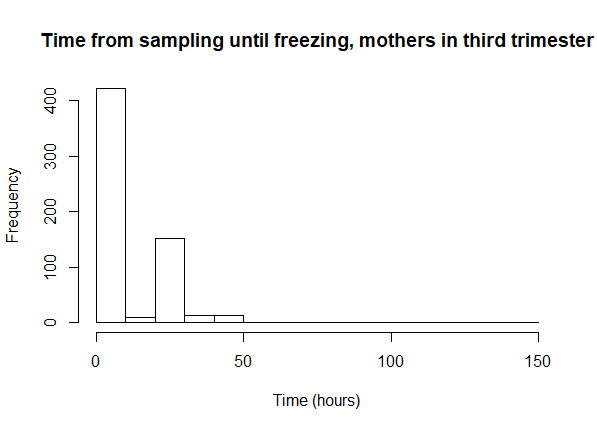


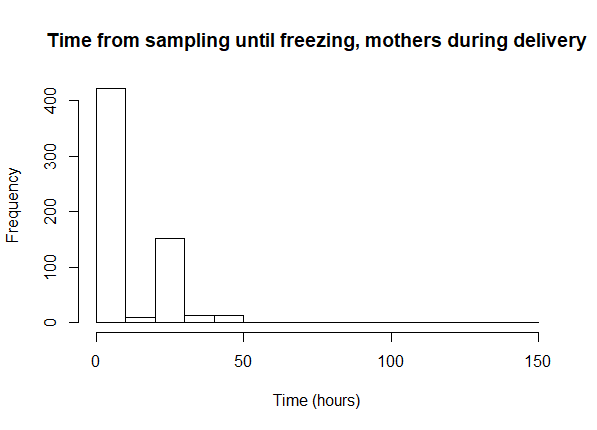


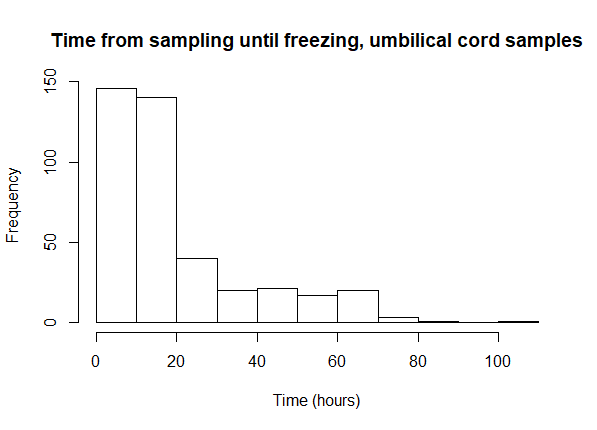


**Figure S3, S4 and S5:** Distributions of time until samples were centrifuged and subsequently frozen in -80° freezer

**Table S1.** Instrument settings for the LC-MS analysis.

| LC and MS settings | |
| --- | --- |
| **Setting/equipment** | **Positive ionization** |
| Column | UPLC HSS T3 (1.8 μm, 2.1 × 100 mm, Waters) |
| gradient | 5%MeOH-95% water, ramped up to 100 % MeOH over 6 min and held for 4.5 min. Both solutions with 0.04 % formic acid |
| Sample volume | 3 µL (positive ionization) 6 µL (negative ionization) |
| Column temperature | 45 °C |
| Flow rate | 0.4 mL/min |
| Ionization source | Dual ESI |
| Scanning range | 50-1700 m/z |
| Scanning rate | 1.67 spectra/second |
| Capilary voltage | 3500 V |
| Gas temperature | 175 °C |
| Gas flow | 10 L/min |
| Nebulizer | 45 psig |

**Table S2.** Final parameters for preprocessing using xcms and RAMClust.

| XCMS peak picking parameters | | |
| --- | --- | --- |
| **Positive ionization** | **Negative Ionization** | **Optim** |
| Method=centWave | Method=centWave | Manual |
| peakwidth=c(6.31,78.15) | peakwidth=c(7.2416,51.15) | IPO |
| prefilter=c(3,3000) | prefilter=c(3,500) | Manual |
| noise=500 | noise=80 | Manual |
| mzdiff=0.0022461 | Mzdiff=0.0039599 | IPO |
| ppm=30.85 | ppm=17.1 | IPO |
| snthresh=10 | snthresh=10 | Manual |
| XCMS retention time adjustment parameters | | |
| Method=Obiwarp | Method=Obiwarp | Manual |
| binsize=0.8 | binsize=0.8 | IPO |
| response=12.16 | response=8.2 | IPO |
| gapInit=0.5225 | gapInit=0.7 | IPO |
| gapExtend=2 | gapExtend=2.5 | IPO |
| XCMS peak grouping parameters | | |
| Method=chromatographic peak density | Method=chromatographic peak density | Manual |
| minFraction=0.4 | minFraction=0.4 | Manual |
| bw=2.5 | bw=2.5 | Manual |
| binSize=0.019 | binSize=0.015 | IPO |
| XCMS peak filling parameters | | |
| ppm=20 | ppm=20 | Manual |
| expandMz=0.25 | expandMz=0.25 | Manual |
| fixedRt=4.76265 | fixedRt=3.57242 | Manual |
| RAMClust parameters | | |
| maxt=5 | maxt=5 | Manual |
| sr=0.35 | sr=0.35 | Manual |
| st=1 | st=1 | Manual |

**Table S3**. Number of metabolomic features per LC-MS mode at key steps during preprocessing.

| **Step** | **RP- Neg** | **RP- Pos** |
| --- | --- | --- |
| XCMS | 1566 | 6961 |
| BatchCorr | 1488 | 6288 |
| RAMClustR | 789 | 3296 |

**Table S4.** Antibodies used in the flow cytometry analysis

| **Cellpopulation studied** | **Monoclonal antibody** | **Fluorochrome** | **Clone** | **Manufacture** |
| --- | --- | --- | --- | --- |
| TruCount  (cell numbers) | anti-CD45  anti-CD4  anti-CD8  anti-CD20 | FITC  PerCP  PE  APC | HI30  SK3  SK1  L27 | BD Bioscience  BD Bioscience  BD Bioscience  BD Bioscience |
| Transitional B-cells | anti-CD20  anti-CD24  anti-CD38  anti-CD5 | PerCP  AF647  PE  FITC | L27  ML5  HB7  UCHT2 | BD Bioscience  BD Bioscience  BD Bioscience  BD Bioscience |
| memory B-cells | anti-CD20  anti-CD27  anti-IgD  anti-IgM | PerCP  FITC  PE  APC | L27  L128  IA6-2  G20-127 | BD Bioscience  BD Bioscience  BD Bioscience  BD Bioscience |
| Recent thymic emigrants | anti-CD4  anti-CD31  anti-CD45RA  anti-CD127 | PerCP  PE  APC  FITC | SK3  WM59  HI100  HIL-7R-M21 | BD Bioscience  BD Bioscience  BD Bioscience  BD Bioscience |
| Naïve and memory T cells | anti-CD4  anti-CD8  anti-CD45RA  anti-CCR7  anti-CD44 | PerCP  PerCP  APC  PE  FITC | SK3  SK1  HI100  G043H7  IM7 | BD Bioscience  BD Bioscience  BD Bioscience  BioLegend  BioLegend |

Abbreviations: APC, Allophycocyanin; PerCp, Peridinin Cholorophyll Protein Complex; FITC, fluorescein isothiocyanate; and PE, R-Phycoerythrin

**Table S5.** All subpopulations of B- and T-cells investigated presented together with number of samples and Q2 from each multivariate model.

|  | **Q2 (cord)** | **n (cord)** | **Q2 (Delivery)** | **n (Delivery)** | **Q2 (Pregnancy)** | **n (Pregnancy)** |
| --- | --- | --- | --- | --- | --- | --- |
| **CD20+ B-cells/ml** Number at birth  Number at 48h  Number at 1m    % at 1m  Number at 4m  % at 4m | -0.141 | 91 | -0.115 | 118 | -0.083 | 115 |
|  | -0.257 | 41 | -0.035 | 57 | -0.154 | 53 |
|  | 0.074 | 60 | -0.165 | 80 | -0.071 | 76 |
|  | -0.338 | 52 | -0.149 | 67 | -0.079 | 77 |
|  | -0.124 | 77 | -0.062 | 105 | -0.085 | 100 |
|  | -0.054 | 60 | -0.074 | 90 | -0.129 | 104 |
| **CD5+ of CD20+ B-cells/ml**  Number at birth  % at birth    Number of at 48h  % at 48h  % at 1m  Number at 4m  % at 4m | -0.050 | 65 | -0.105 | 79 | -0.067 | 76 |
|  | -0.150 | 93 | -0.198 | 112 | -0.048 | 108 |
|  | 0.107 | 21 | -0.109 | 30 | -0.128 | 29 |
|  | -0.383 | 29 | -0.235 | 48 | -0.034 | 47 |
|  | -0.253 | 47 | 0.014 | 60 | -0.085 | 70 |
|  | 0.228 | 22 | -0.604 | 26 | -0.765 | 25 |
|  | -0.280 | 59 | -0.160 | 86 | -0.142 | 100 |
| **CD24hiCD38low Memory of CD20+ B-cells/ml**  Number at birth  % at birth  % at 48h  % at 1m  Number at 4m  % at 4m | 0.024 | 65 | -0.162 | 79 | -0.110 | 76 |
|  | -0.057 | 93 | -0.047 | 112 | -0.040 | 108 |
|  | -0.030 | 21 | -0.128 | 38 | -0.499 | 44 |
|  | -0.228 | 47 | -0.055 | 60 | 0.110 | 70 |
|  | 0.235 | 22 | 0.208 | 26 | -0.106 | 25 |
|  | 0.066 | 59 | -0.000 | 86 | -0.170 | 100 |
| **CD24lowCD38low Naive of CD20+ B-cells/ml** Number at birth  % at birth  % at 48h | -0.011 | 65 | -0.050 | 79 | -0.057 | 76 |
|  | -0.111 | 93 | -0.067 | 112 | -0.094 | 108 |
|  | -0.328 | 21 | -0.185 | 38 | 0.015 | 44 |
| **CD24+CD38+ Naive B-cells** % at 1m  Number at 4m  % at 4m | -0.090 | 47 | -0.117 | 60 | -0.154 | 70 |
|  | -0.278 | 22 | -0.063 | 26 | -0.122 | 25 |
|  | -0.163 | 59 | -0.174 | 86 | -0.243 | 100 |
| **CD24hiCD38hi Transitional of CD20+ B-cells** Number at birth  % at birth  % at 48h  % at 1m  Number at 4m  % at 4m | -0.059 | 65 | -0.006 | 79 | -0.008 | 76 |
|  | -0.040 | 93 | -0.165 | 112 | -0.049 | 108 |
|  | -0.160 | 21 | -0.042 | 38 | 0.097 | 44 |
|  | -0.137 | 47 | -0.062 | 60 | -0.121 | 70 |
|  | -0.496 | 22 | -0.012 | 26 | -0.087 | 25 |
|  | -0.303 | 59 | -0.100 | 86 | -0.146 | 100 |
| **class-switched of CD20+ B-cells/ml** Number at birth  % at birth  % at 48h  % at 1m  Number at 4m  % at 4m | -0.101 | 69 | -0.140 | 87 | -0.027 | 84 |
|  | -0.205 | 98 | -0.094 | 121 | -0.050 | 116 |
|  | -0.565 | 24 | -0.252 | 44 | -0.174 | 50 |
|  | 0.032 | 52 | 0.012 | 67 | -0.025 | 77 |
|  | 0.067 | 21 | -0.027 | 25 | -0.062 | 24 |
|  | -0.137 | 60 | -0.167 | 90 | -0.078 | 104 |
| **IgDhi IgMlow of B-cells/ml**  Number at birth  % at birth  % at 48h  % at 1m  Number at 4m  % at 4m | 0.024 | 69 | -0.077 | 87 | -0.151 | 84 |
|  | -0.131 | 98 | -0.067 | 121 | -0.019 | 116 |
|  | -0.435 | 24 | -0.216 | 44 | -0.369 | 50 |
|  | -0.258 | 52 | -0.112 | 67 | -0.052 | 77 |
|  | -0.0186 | 21 | -0.347 | 25 | -0.334 | 24 |
|  | -0.100 | 60 | -0.183 | 90 | -0.063 | 104 |
| **IgM+ memory B-cells**  % at 1m  Number at 4m  % at 4m | 0.048 | 52 | -0.160 | 67 | -0.093 | 77 |
|  | -0.178 | 21 | -0.782 | 25 | -0.238 | 24 |
|  | -0.304 | 60 | -0.115 | 90 | -0.165 | 104 |
| **CD27+ Memory of CD20+ B-cells/ml** Number at birth  % at birth  % at 48h  % at 1m  Number at 4m  % at 4m | -0.124 | 69 | -0.080 | 87 | -0.070 | 84 |
|  | -0.182 | 98 | -0.039 | 121 | -0.133 | 116 |
|  | -0.438 | 24 | 0.123 | 44 | -0.251 | 50 |
|  | -0.169 | 52 | -0.076 | 67 | -0.045 | 77 |
|  | -0.340 | 21 | 0.074 | 25 | 0.211 | 24 |
|  | -0.123 | 60 | -0.098 | 90 | -0.213 | 104 |
| **IgDlow IgMhi of CD20+ B-cells/ml** Number at birth  % at birth  % at 48h | -0.030 | 69 | -0.199 | 87 | -0.115 | 84 |
|  | -0.087 | 98 | -0.157 | 121 | -0.057 | 116 |
|  | -0.004 | 24 | -0.071 | 44 | -0.219 | 50 |
| **CD4+ T-cells/ml** Number at birth  Number at 48h  Number at 1m  Number at 4m | -0.144 | 100 | -0.153 | 127 | -0.129 | 124 |
|  | -0.179 | 41 | -0.069 | 57 | -0.089 | 53 |
|  | -0.115 | 61 | -0.227 | 81 | -0.196 | 77 |
|  | -0.087 | 77 | -0.183 | 105 | -0.097 | 100 |
| **CD31-CD45RA+ of CD4+ T-cells/ml** Number at birth  % at birth  Number at48h  % at 48h  Number at 1m  % at 1m  Number at 4m  % at 4m | -0.171 | 46 | -0.151 | 62 | -0.337 | 61 |
|  | -0.140 | 70 | -0.219 | 95 | 0.050 | 92 |
|  | -0.171 | 30 | -0.110 | 42 | -0.068 | 41 |
|  | -0.017 | 47 | -0.079 | 74 | -0.145 | 72 |
|  | -0.070 | 56 | -0.266 | 75 | -0.148 | 72 |
|  | 0.001 | 65 | -0.113 | 93 | -0.212 | 89 |
|  | -0.207 | 66 | -0.070 | 89 | 0.066 | 86 |
|  | -0.226 | 75 | -0.222 | 104 | -0.151 | 101 |
| **CD31+CD45RA+ of CD4+ T-cells/ml** Number at birth  % at birth  Number at 48h  % at 48h  Number at 1m  % at 1m  Number at 4m    % at 4m | -0.097 | 46 | -0.152 | 62 | -0.512 | 61 |
|  | -0.075 | 70 | -0.071 | 95 | -0.199 | 92 |
|  | 0.031 | 30 | -0.154 | 43 | -0.053 | 42 |
|  | 0.001 | 47 | -0.155 | 74 | -0.122 | 72 |
|  | -0.103 | 56 | -0.156 | 75 | -0.065 | 72 |
|  | -0.043 | 65 | -0.038 | 93 | -0.182 | 89 |
|  | -0.147 | 66 | -0.182 | 89 | -0.071 | 86 |
|  | -0.219 | 75 | -0.144 | 104 | -0.041 | 101 |
| **CCR7+CD45RA+ of CD4+ T-cells** Number at birth  % at birth  Number at 48h  % at 48h  Number at 1m  % at 1m  Number at 4m  % at 4m | -0.230 | 47 | -0.082 | 63 | -0.092 | 62 |
|  | 0.152 | 71 | 0.010 | 99 | -0.129 | 95 |
|  | -0.005 | 27 | -0.086 | 40 | 0.076 | 38 |
|  | -0.158 | 40 | -0.098 | 68 | -0.233 | 65 |
|  | -0.264 | 55 | -0.175 | 74 | -0.163 | 71 |
|  | -0.109 | 64 | -0.052 | 91 | -0.080 | 87 |
|  | -0.096 | 66 | -0.126 | 90 | -0.132 | 87 |
|  | -0.101 | 76 | -0.163 | 104 | -0.224 | 101 |
| **CCR7+CD45RA- of CD4+ T-cells** Number at birth  % at birth  Number at 48h  % at 48h  Number at 1m  % at 1m  Number at 4m  % at 4m | -0.140 | 47 | 0.014 | 62 | 0.018 | 61 |
|  | -0.199 | 71 | -0.148 | 99 | -0.121 | 95 |
|  | -0.099 | 27 | -0.310 | 40 | -0.019 | 38 |
|  | 0.037 | 40 | -0.056 | 68 | 0.009 | 65 |
|  | 0.085 | 55 | -0.055 | 74 | 0.014 | 71 |
|  | -0.275 | 64 | -0.044 | 91 | -0.021 | 87 |
|  | 0.063 | 66 | -0.162 | 90 | 0.079 | 87 |
|  | -0.065 | 76 | -0.001 | 104 | 0.002 | 101 |
| **CCR7-CD45RA- of CD4+ T-cells** Number of birth  % at birth  Number at 48h  % at 48h  Number at 1m  % at 1m  Number at 4m  % at 4m | -0.096 | 47 | -0.111 | 63 | -0.318 | 62 |
|  | -0.147 | 71 | -0.104 | 99 | -0.179 | 95 |
|  | -0.071 | 27 | -0.228 | 40 | -0.447 | 38 |
|  | -0.102 | 40 | -0.070 | 68 | -0.023 | 65 |
|  | 0.068 | 55 | -0.172 | 74 | -0.013 | 71 |
|  | -0.153 | 64 | -0.138 | 91 | -0.139 | 87 |
|  | -0.135 | 66 | -0.026 | 89 | -0.170 | 86 |
|  | 0.031 | 76 | -0.103 | 104 | -0.189 | 101 |
| **CCR7-CD45RA+ of CD4+ T-cells** Number at 48h  % at 48h  Number at 1m  % at 1m  Number at 4m  % at 4m | -0.108 | 27 | -0.192 | 40 | -0.111 | 38 |
|  | -0.152 | 40 | -0.434 | 68 | -0.108 | 65 |
|  | -0.324 | 55 | 0.039 | 74 | -0.159 | 71 |
|  | -0.128 | 64 | -0.201 | 91 | -0.097 | 87 |
|  | -0.073 | 66 | -0.177 | 90 | -0.215 | 87 |
|  | -0.167 | 76 | -0.077 | 104 | -0.076 | 101 |
| **CD8+ T-cells/ml** Number at birth  Number at 48h  Number atl 1m  Number at 4m | -0.067 | 100 | -0.065 | 127 | -0.068 | 124 |
|  | -0.144 | 41 | -0.177 | 57 | -0.136 | 53 |
|  | -0.024 | 61 | -0.200 | 81 | -0.231 | 77 |
|  | -0.010 | 77 | -0.119 | 105 | -0.093 | 100 |
| **CCR7-CD45RA+ of CD8+ T-cells** Number at birth  % at birth  Number at 48h  % at 48h  Number at 1m  % at 1m  Number at 4m  % at 4m | 0.154 | 46 | -0.044 | 63 | -0.143 | 62 |
|  | 0.048 | 68 | -0.252 | 93 | -0.222 | 89 |
|  | -0.242 | 23 | -0.174 | 35 | -0.243 | 33 |
|  | -0.302 | 36 | -0.333 | 61 | -0.055 | 58 |
|  | -0.160 | 54 | -0.150 | 71 | -0.155 | 68 |
|  | -0.057 | 62 | -0.050 | 87 | -0.047 | 83 |
|  | -0.042 | 66 | -0.098 | 89 | -0.076 | 86 |
|  | -0.100 | 77 | -0.093 | 104 | -0.062 | 101 |
| **CCR7+CD45RA+ of CD8+ T-cells** Number at birth  % at birth  Number at 48h  % at 48h  Number at 1m  % at 1m  Number at 4m  % at 4m | -0.307 | 46 | 0.160 | 63 | -0.054 | 62 |
|  | -0.004 | 68 | -0.243 | 93 | -0.113 | 89 |
|  | -0.134 | 23 | -0.144 | 35 | -0.109 | 33 |
|  | -0.397 | 36 | -0.289 | 61 | -0.104 | 58 |
|  | -0.056 | 54 | 0.089 | 71 | -0.160 | 68 |
|  | -0.086 | 62 | -0.132 | 87 | 0.013 | 83 |
|  | -0.080 | 66 | -0.200 | 89 | -0.167 | 86 |
|  | -0.062 | 77 | -0.210 | 104 | -0.222 | 101 |
| **CCR7+CD45RA- of CD8+ T-cells** Number at birth  % at birth  Number at 48h  % at 48h  Number at 1m  % at 1m  Number at 4m  % at 4m | -0.186 | 46 | -0.184 | 62 | -0.133 | 61 |
|  | -0.115 | 68 | -0.097 | 93 | -0.073 | 89 |
|  | -0.028 | 23 | -0.216 | 35 | -0.118 | 33 |
|  | -0.107 | 36 | 0.041 | 61 | -0.113 | 58 |
|  | 0.029 | 52 | -0.048 | 68 | -0.081 | 65 |
|  | -0.129 | 62 | 0.005 | 87 | -0.121 | 83 |
|  | -0.074 | 66 | -0.192 | 89 | -0.092 | 86 |
|  | -0.091 | 77 | 0.000 | 104 | -0.002 | 101 |
| **CCR7-CD45RA- of CD8+ T-cells** Number at birth  % at birth  Number at 48h  % at 48h  Number at 1m  % at 1m  Number at 4m  % at 4m | -0.318 | 46 | -0.174 | 62 | -0.182 | 61 |
|  | -0.054 | 68 | -0.202 | 93 | -0.113 | 89 |
|  | 0.045 | 23 | -0.127 | 35 | -0.234 | 33 |
|  | -0.424 | 36 | 0.005 | 61 | -0.137 | 58 |
|  | -0.081 | 54 | -0.048 | 70 | -0.076 | 67 |
|  | -0.105 | 62 | -0.114 | 87 | -0.042 | 83 |
|  | -0.066 | 66 | -0.079 | 89 | -0.150 | 86 |
|  | 0.066 | 77 | -0.139 | 104 | -0.251 | 101 |

**Table S6.** Parameter settings used for multivariate modeling using the MUVR algorithm.

| Parameter | Value |
| --- | --- |
| nOuter | 8 |
| nInner | 7 |
| varRatio | 0.9 |
| nRep | 20 |

**Table S7.** List of metabolites previously found to be associated with manifest allergy.

| Metabolite |
| --- |
| (ent-2b,4S,9a)- 2,4,9-Trihydroxy- 10(14)-oplopen-3- one 2-(2- methylbutanoate) 9- (3-methyl-2E- pentenoate) |
| C50:1 TAG |
| (2E,6E)-2,6- Nonadienal |
| 1-(1-enyl-oleoyl)-GPE (P-18:1) |
| 1-(1-enyl-palmitoyl)-2-oleoyl-GPC (P-16:0/18:1) |
| 1-(1-enyl-palmitoyl)-2-palmitoyl-GPC (P-16:0/16:0) |
| 1-(1-enyl-stearoyl)-GPE (P-18:0) |
| 1,2-dipalmitoyl-GPC (16:0/16:0) |
| 1-arachidonoyl-GPE (20:4n6) |
| 1-docosapentaenoylglycerophosphocholine (22:5) |
| 1-linolenoyl-GPC (18:3) |
| 1-linoleoyl-2-arachidonoyl-GPC (18:2/20:4n6) |
| 1-linoleoyl-GPC (18:2) |
| 1-Methoxy-1H-indole-3- carboxaldehyde |
| 1-myristoyl-2-arachidonoyl-GPC (14:0/20:4) |
| 1-myristoyl-2-arachidonoyl-GPC (14:0/20:4) |
| 1-myristoyl-2-palmitoyl-GPC (14:0/16:0) |
| 1-palmitoleoyl-GPC (16:1) |
| 1-palmitoyl-2-stearoyl-GPC (16:0/18:0) |
| 1-palmitoyl-GPC (16:0) |
| 1-stearoyl-2-arachidonoyl-GPI (18:0/20:4) |
| 2,2,4,6,6-pentamethylheptane |
| 2-aminoadipate |
| 2-hydroxy-2-methylbutanedioic acid |
| 2-hydroxybutyrate |
| 2-hydroxyisobutyrate |
| 2-isopropylmalic acid |
| 2-oxobutanoate |
| 3-aminoisobutyrate |
| 3-hydroxy-3-methylglutarate |
| 3-hydroxyhexanoate |
| 3-methyladipate |
| 3-methylglutaconate |
| 4-hydroxyphenylpyruvate |
| 5,6-dihydrothymine |
| 6-phospho-d-gluconate |
| acetylcarnitine |
| Adenine |
| alanine |
| alpha-ketoglutaric acid |
| arginine |
| ascorbic acid |
| Aspartic acid |
| behenoyl dihydrosphingomyelin (d18:0/22:0) |
| Benzoic acid |
| Betaine |
| Biliverdin |
| C36:4 DAG |
| C44:0 PG |
| C4-OH carnitine |
| C56:1 TAG |
| Calystegine A6 |
| caproate (6:0) |
| Cellobiose |
| ceramide (d16:1/24:1, d18:1/22:1) |
| ceramide (d18:1/20:0, d16:1/22:0, d20:1/18:0) |
| cholesterol |
| Cholic acid |
| choline |
| cis-aconitate |
| citrate |
| CMP |
| Cortisone |
| Creatine |
| creatinine |
| decanal |
| decane |
| Delta-tocopherol |
| deoxyadenosine |
| d-glucarate |
| dimethylarginine |
| dimethylglycine |
| dodecanal |
| dodecane |
| Docosapentaenoic acid |
| Eicosapentaenoic acid |
| fumarate |
| gamma-glutamylalanine |
| gamma-glutamylthreonine |
| gamma-linolenic acid |
| gamma-tocopherol/beta-tocopherol |
| glucose |
| glutamate |
| glutamine |
| Glutathione (red) |
| Glyceric acid |
| glycerophosphoinositol |
| glycine |
| glycocholate |
| Glycolithocholate |
| glycosyl-N-stearoyl-sphingosine (d18:1/18:0) |
| Glycoursodeoxycholate |
| heptenedioate (C7:1-DC) |
| Hexadacanoic acid |
| hexose-phosphate |
| histidine |
| Hydroxyphenylacetic acid |
| Hypoxanthine |
| Isocitric acid |
| isoleucine |
| lactate |
| Lactose |
| lactosyl-N-nervonoyl-sphingosine (d18:1/24:1) |
| lactosyl-N-palmitoyl-sphingosine (d18:1/16:0) |
| leucine |
| lignoceroyl sphingomyelin (d18:1/24:0) |
| lysine |
| maleic acid |
| Methanol |
| Methionine |
| Methyl-imidazoleacetic acid |
| Methylnicotinamide |
| myristic acid |
| myristoyl dihydrosphingomyelin (d18:0/14:0) |
| N1-Methyl-2-pyridone-5-carboxamide |
| N2-acetyllysine |
| N-acetylhistidine |
| N-acetylleucine |
| N-acetylmethionine |
| N-acetylneuraminate |
| N-acetylornithine |
| N-acetylthreonine |
| N-acetylvaline |
| N-formylmethionine |
| Nigerose |
| nonanal |
| Nonane |
| Norleucine |
| octadecanedioate (C18-DC) |
| Octadecenoic acid |
| oleic acid |
| oleoyl-arachidonoyl-glycerol (18:1/20:4) |
| Ophthalmic acid |
| orotate |
| Oxoisocaproic acid |
| palmitoleic acid |
| palmitoyl dihydrosphingomyelin (d18:0/16:0) |
| palmitoyl sphingomyelin (d18:1/16:0) |
| palmitoyl-arachidonoyl-glycerol (16:0/20:4) |
| P-cresol sulfate |
| Proline betaine |
| Putrescine |
| Pyroglutamic acid |
| Pyrophosphate |
| Retinoic acid |
| sarcosine |
| shikimate-3-phosphate |
| Sorbitol |
| sphingomyelin (d17:1/16:0, d18:1/15:0, d16:1/17:0) |
| sphingomyelin (d18:0/20:0, d16:0/22:0) |
| sphingomyelin (d18:1/14:0, d16:1/16:0) |
| sphingomyelin (d18:1/22:1, d18:2/22:0, d16:1/24:1) |
| sphingomyelin (d18:1/22:2, d18:2/22:1, d16:1/24:2) |
| sphingomyelin (d18:2/14:0, d18:1/14:1) |
| sphingomyelin (d18:2/16:0, d18:1/16:1) |
| sphingomyelin (d18:2/21:0, d16:2/23:0) |
| sphingomyelin (d18:2/23:1) |
| Sphingomyelin (d18:2/23:0, d18:1/23:1, d17:1/24:1) |
| Sphingosine 1 phosphate |
| stearoyl-arachidonoyl-glycerol (18:0/20:4) |
| stearoylcarnitine (C18) |
| succinate |
| taurochenodeoxycholate |
| Taurocholate |
| Taurocyamine |
| taurodeoxycholate |
| Tauroursodeoxycholate |
| tetradecane |
| Thiamine |
| threonine |
| trehalose-6-phosphate |
| tricosanoyl sphingomyelin (d18:1/23:0) |
| Tryptophan |
| Tryptophan Betaine |
| tyrosine |
| Uracil |
| Uric acid |
| Uridine |
| Urocanic acid |
| Ursodeoxycholic acid |
| valine |
| Vitamin D |


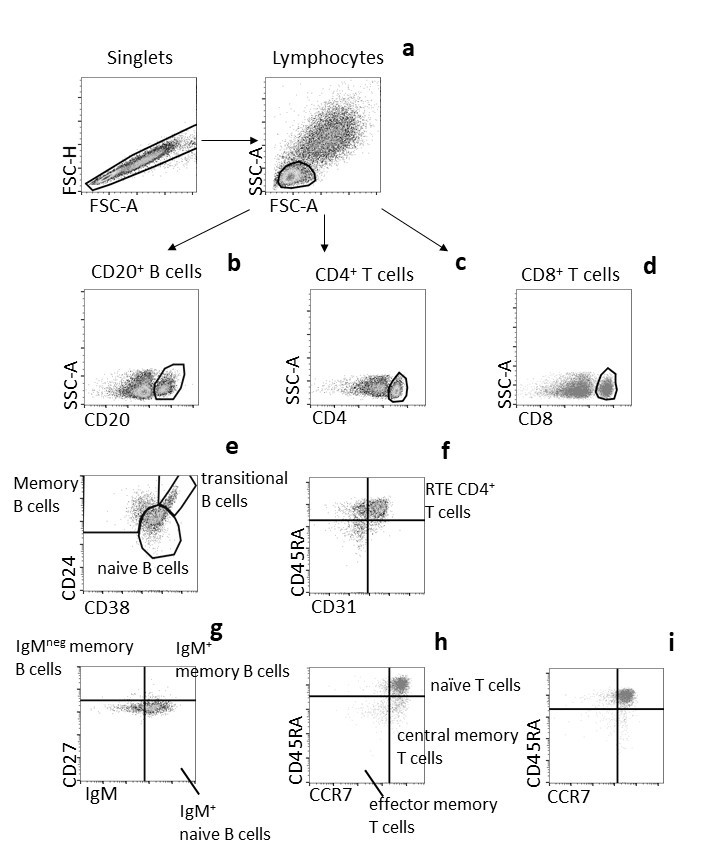


**Figure S6. Gating strategy describing the different subsets of CD20^+^ B cells, CD4^+^ and CD8^+^ T cells**. (a) Singlet PBMC were gated for lymphocytes, which were further gated for CD20^+^ B cells (b), CD4^+^ (c) or CD8^+^ (d) T cells. (e) The B cells were subdivided into CD24^hi^CD38^hi^ transitional, CD24^low^CD38^low^ naïve and CD24^+^CD34^neg^ memory B cells. (f) With the use CD27 and IgM, B cells were also subdivided into IgM^+^CD27^neg^ naïve, IgM^+^CD27^+^ memory B cells and IgM^neg^ CD27^+^ memory B cells. (g) Recent thymic emigrants were identified within the CD4^+^ T cells as CD31^+^CD45RA^+^ cells. (g-i) Naïve T cells, central memory and effector memory T cells were identified in the CD4^+^ (h) and CD8^+^ T cells (i), with the use of the surface markers CD45RA and CCR7.

## Supplementary methods

The Q2 value is defined as 1 – predictive residual error sum of squares (PRESS) from the test data divided by the total sum of squares (TSS) in the training data. With TSS and PRESS defined as the following:

 TSS = ${\sum_{i=1}^{n} (y-\bar{y})}^{2}$

 PRESS = ${\sum_{i=1}^{m} (y- ŷ)}^{2}$

Q2 = $1-\frac{PRESS}{TSS}$

Classification rate is calculated as:
(Number of correctly classified samples)/(Total number of samples)*100
